# A Constrained Coding-Aware Routing Scheme in Wireless Ad-Hoc Networks

**DOI:** 10.3390/s19102252

**Published:** 2019-05-15

**Authors:** Yimin Zhao, Song Xiao, Hongping Gan, Lizhao Li, Lina Xiao

**Affiliations:** State Key Lab of Integrated Services Networks, School of Communication Engineering, Xidian University, Xi’an 710071, China; zhaoyimin@stu.xidian.edu.cn (Y.Z.); uqhgan@uq.edu.au (H.G.); lz_li@stu.xidian.edu.cn (L.L.); linaxiao@stu.xidian.edu.cn (L.X.)

**Keywords:** network coding, coding-aware routing, coding conditions, coding benefit, wireless ad-hoc networks

## Abstract

In wireless multi-hop networks, instead of using the traditional store-and-forward method, the relay nodes can exploit the network coding idea to encode and transmit the packets in the distributed coding-aware routing (DCAR) mechanisms, which can decrease the transmission number and achieve higher throughput. However, depending on the primary coding conditions of DCAR, the DCAR-type schemes may not only detect more coding opportunities, but also lead to an imbalanced distribution of the network load. Especially, they are not energy efficient in more complex scenarios, such as wireless ad-hoc networks. In this paper, to solve these shortcomings, we propose a constrained coding-aware routing (CCAR) mechanism with the following benefits: (1) by the constrained coding conditions, the proposed mechanism can detect good coding opportunities and assure a higher decoding probability; (2) we propose a tailored “routing + coding” discovery process, which is more lightweight and suitable for the CCAR scheme; and (3) by evaluating the length of the output queue, we can estimate the states of coding nodes to improve the efficient coding benefit. To those ends, we implement the CCAR scheme in different topologies with the ns-2 simulation tool. The simulation results show that a higher effective coding benefit ratio can be achieved by the constrained coding conditions and new coding benefit function. Moreover, the CCAR scheme has significant advantages regarding throughput, average end-to-end delay, and energy consumption.

## 1. Introduction

**Figure 1:**
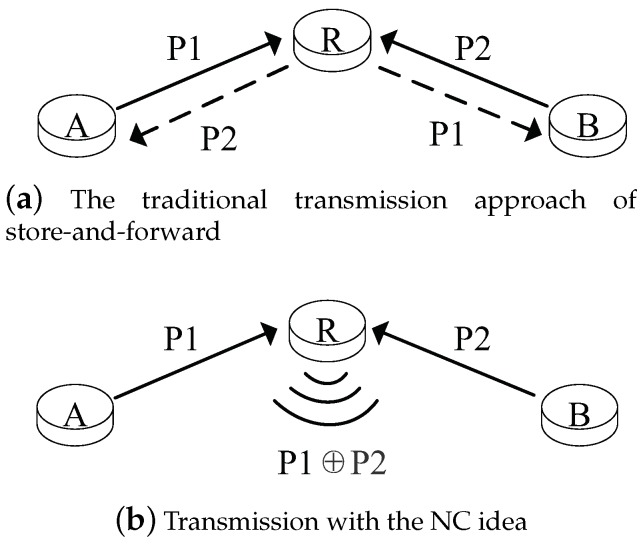
A simple example of the network coding (NC) idea.

Over the past few years, given the increased requirements of the network throughput and delay in wireless networks, network coding (NC), proposed by Ahlswede et al., has received much attention [1]. NC can achieve the upper limit of the maximum transmission flow. Compared with the traditional store-and-forward transmission method, NC allows for the intermediate nodes to mix some packets from different flows and broadcast the encoded packets to the receivers. Figure 1 illustrates a simple example of the NC idea, where Nodes A and B need to exchange some data via the intermediate Node R. The traditional store-and-forward method is shown in Figure 1a. After the intermediate Node R receives the Packet P1 (P2) from Node A (B), it will transmit Packet P1 (P2) to Node B (A). The transmission counts are four times in this process, and it may not be the best choice. Now, considering the transmission with the NC idea in Figure 1b, Node A (B) sends its respective Packet P1 (P2) to intermediate Node R. Then, Node R has a coding opportunity and can encode the two packets with an exclusive or (XOR) operation and broadcast the encoded Packet P1⊕P2. Nodes A and B can recover the encoded packet by their own packet with a simple XOR operation again. The transmission counts are only three times in this NC-based transmission process, and the reduced transmission counts can save energy. NC is more useful for wireless networks because it can explore the broadcast nature of wireless links and reduce the negative characteristics (e.g., interference, channel fading, and energy constraints) [2,3]. Moreover, the NC technology can be also widely applied to numerous aspects, such as distributed storage, cognitive radio, communication security, and P2P streaming system [4].

Not surprisingly, the NC technology and routing protocols can be combined. The first practical NC-based routing scheme, called COPE, was proposed by Katti et al. [5]. They combined the routing protocol and network coding in a real network test-bed that can detect the coding opportunities and increase the network performance obviously [6]. However, the COPE-like schemes have some disadvantages: for example, their coding opportunities depend on certain topologies and limits in the two-hop region [7]. For more flexible detection of the coding opportunities, some researchers have combined the NC and opportunistic routing (OR) technologies, termed “coding-aware routing (CAR)”. In the CAR scheme, the relay nodes can be aware of all the information of one-hop neighbors and flows and can then judge whether to encode according to the link quality or other information [8]. However, the traditional CAR scheme may induce more overhead, and the coding opportunities will concentrate on the part of flows [9]. Then, based on CAR, J.Le et al. proposed a distributed coding-aware routing (DCAR) scheme [10]. Because the candidate paths are diverse and have no fixed route in wireless multi-hop networks [11], the DCAR scheme introduced the primary coding conditions (PCC) to detect all possible coding opportunities in all candidate paths, which are more suitable for wireless multi-hop networks. The simulation results showed that the DACR scheme has much higher throughput than the original CAR and COPE-like schemes. Then, based on PCC, more DACR-type schemes emerged in large numbers [12,13,14,15]. B. Guo et al. proposed a free-ride-oriented routing metric to make PCC more implementable [12]. J. Chen et al. improved the routing decision process to suit PCC and ensure a higher decoded ratio [14,15]. L. F. Xie et al. applied a consistency of encoding and overhearing (CEO) principle to the DCAR scheme to guarantee successful decoding of all encoded or re-encoded packets [16]. In addition, although the DCAR-type schemes have more coding opportunities and larger network throughput by PCC, they will cause imbalanced loads, for which the encoded and decoding nodes will coincide or be closed.

To overcome the shortcomings of the DCAR-type schemes, we propose a constrained coding-aware routing (CCAR) mechanism, which is more energy-efficient and suitable for wireless ad-hoc networks. The CCAR mechanism aims to find a more appropriate route to maximize the coding benefits instead of the coding opportunities. We also modify the routing discovery process to ensure the balance of the network load. Moreover, the proposed scheme cannot only save more energy, but can also encode the flows with different rates. The contributions of this paper can be summarized as follows:

1. To reduce network congestion and balance network load, we propose the constrained coding conditions (CCC) modified from PCC, which allows some neighbor nodes of the destination nodes to assist in decoding.
2. To save energy and be more adaptive to the constrained coding conditions, the RREQ and RREP messages are revised. Moreover, we combine and simplify the routing discovery and coding processes.
3. We use the length of the output queue as an indicator of the current status of the coding nodes and propose a novel coding benefit function to reduce the negative effects of rate diversity.

The results show that our scheme achieves better network performance with respect to network throughput, delay, and energy consumption.

The remainder of the paper is organized as follows. Section 2 reviews the related work concerning the coding-aware routing mechanism. Section 3 presents the routing ideas, procedures, and implementation issues of CCAR. Section 4 evaluates the performance of the CCAR scheme through the simulation results. Conclusions are drawn in Section 5.

## 2. Related Work

Concerning the routing protocols applied in wireless ad-hoc networks, the first attempt was the destination-sequenced distance-vector (DSDV) routing protocol, which was based on the Bellman–Ford algorithm [17]. By evaluating and utilizing the link state, P. Jacquet et al. proposed an optimized link state routing (OLSR) protocol to find a route with a better link actively [18]. However, these routing protocols, which belong to a proactive work style, may induce more energy consumption. Some routing protocols focus on a reactive work style. C. E. Perkins proposed an ad-hoc on-demand distance vector (AODV) routing protocol with a lower network overhead and network utilization [19]. To adapt to the changes in the network topology, dynamic source routing (DSR) was proposed by D. B. Johnson [20]. Moreover, OR and CAR are also attributable to this type of routing [8].

CAR can apply to different wireless multi-hop networks [21], such as wireless mesh networks [22], wireless ad-hoc networks [23], and wireless sensor networks [24]. The CAR schemes are often classified into two categories. One is called intra-flow coding (IAC) routing, which can encode the original packets that belong to the same flow [25]. Paul-Louis Ageneau et al. proposed a constraint-aware IAC scheme to enhance reliability [26]. The other is inter-flow coding (IRC) routing, which can encode the original packets from different flows. The work in [27] combined the IAC and IRC methods to improve the robust performance of lossy wireless networks. However, the IAC method is more complicated than the IRC method because it will consume more time slots to send different data to different destinations. In this paper, we only focus on the IRC routing mechanisms. In these mechanisms, COPE [6] is the first IRC routing mechanism applied to practical wireless network protocols. COPE is also compatible with existing network protocols and algorithms. It has a better performance than traditional store-and-forward transmission methods using TCP and UDP. Many other derivatives have appeared. However, the COPE-like schemes have limitations such that the analyses are often in regular topologies, such as the Alice-Bob topology, X-topology, Y-topology, and star topology.

Then, to make the CAR schemes no longer dependent on regular topologies, Le et al. [10] proposed a distributed solution, the DCAR scheme, to increase the ability of detecting the coding opportunities. Based on PCC, the coding opportunities can be carefully found in all possible routes. Compared to COPE, DCAR obtains better performance in terms of the network throughput and delay. Many researchers used the DCAR idea to design different routing schemes for a more complex wireless network. However, choosing when, where, and which packets to encode in wireless networks is an NP-hard problem [28]. Moreover, PCC may cause partial network congestion in a more complex wireless network, in which the decoding nodes are also the coding nodes or the decoding nodes are too close to the coding nodes. To avoid network congestion, some DCAR-type schemes using different metrics have been proposed. B. Guo et al. proposed a free-ride-oriented routing metric (FORM), which can more accurately identify and utilize good coding opportunities [12]. To reduce the confusion of the coding opportunities, J. Chen et al. revised the routing discovery procedures in the DCAR scheme by using the four steps of RREQ (route request), RREP (route reply), RCON (routing confirm), and RACK (routing acknowledge) [15]. To overcome network dynamics (e.g., high packet loss rate and node mobility), Minhae Kwon and Hyunggon Park proposed an NC-based evolutionary network formation to adapt to the topology change [29]. Shao Xing et al. proposed a load-balanced coding-aware routing metric that considered the load degree of each node, and the traffic of the scheme was evenly spread [30]. They also introduced a traffic-shaped CAR to increase the actual occurrences of network coding for wireless sensor networks [31].

Furthermore, most CAR and DCAR schemes are based on the idealized assumption that data flows have the same rate. Hence, some researchers focused on the problem of the multi-rate interference in the CAR schemes. The work in [32] proved that NC could improve network throughput in a rate-diversity scenario, and the authors used a new linear programming model to calculate the maximal throughput. By exploiting the spatial diversity, J. Zhang et al. proposed a cooperative network coding (CNC) mechanism to find coding opportunities in wireless multi-rate and -hop networks, and they provided a theoretical formulation to calculate the maximal throughput [33]. L. F. M. Vieira et al. concluded that the rate-matching problem in the coding nodes was among the main influential elements in wireless multi-rate networks [32]. However, these works are theoretical and not easily implemented. Y. Chi et al. proposed a simple implementation that used a weighting factor to react to flow intersection of different rates. The weighting factor consists of the counter reuse ratio and destination address reuse ratio [34]. Afterward, some works focused on the routing protocols considering the queue length in the coding nodes to solve the rate-matching problem [35]. Queue length can react to the node states and is also easy to implement. The DCAR scheme used a simple modified interference queue length (MIQ) to respond to the node state and incorporate traffic load, and the interference information will be gathered in a unified manner [10]. H. Seferoglu and A. Markopoulou used the queue management scheme to improve the TCP performance of CAR [36]. In our past work [37], we used a Markov chain to formulate the queue state of the coding nodes and proposed an OQMCAR algorithm based on the queue state and local topology to reduce the network end-to-end delay. The summarized view of the key related works is shown in Table 1.

In light of the aforementioned works, there has been intense interest in avoiding congestion in wireless multi-flow networks and in theoretically analyzing the rate-matching problem in wireless multi-rate networks. However, the primary coding conditions that cause the congestion seldom change. In this work, we propose the constrained coding conditions to reduce the congestion and use a novel coding benefit function with consideration of the queue length to address the rate-matching problem easily.

## 3. Routing Implementation

In this section, we modify the primary coding conditions to constrain the bad coding opportunities, which was rarely done in previous related works. Then, we illustrate the process of routing discovery, including a new RREQ packet format. Finally, we consider the states of the coding nodes and introduce a novel coding benefit function.

### 3.1. System Model

Before we discuss the details of the novel coding-aware routing implementation, the system model and some crucial terms need to be introduced first. We construct the immovable nodes in a wireless ad-hoc network, which can support multiple multicast and unicast sessions. All nodes are in a promiscuous mode, such that each node can both overhear the data transmission and obtain the delivery probabilities of links with its one-hop neighbors. Each node has a buffer pool that can store the original and encoded packets that are sent and overheard. Moreover, the original or encoded packets in each buffer are used for encoding or decoding. The buffer is large enough to store the packets. The sources and destinations of each flow are randomly and independently selected, and the inter-flow encoding and decoding operations are XOR in this paper. The related CCAR terms are explained in Table 2 below.

### 3.2. Coding Conditions

The coding conditions are essential components in coding-aware routing schemes to determine which nodes have coding opportunities. Then, we first review the primary coding conditions that can detect the coding opportunities on all paths. We will then introduce the constrained coding conditions and describe the difference between the primary and constrained coding conditions in both two-flow and multi-flow networks.

**Figure 2:**
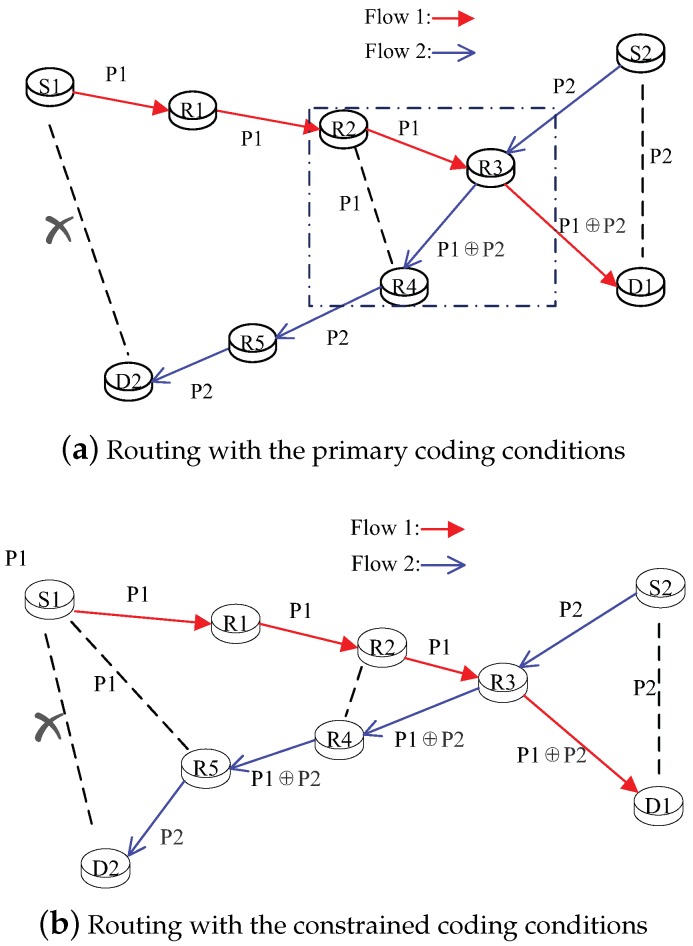
The routing decision with different coding conditions in a two-flow network.

Let *z* denote a node. N(z) denotes the set of each one-hop neighbor of node *z*, and *f* denotes a flow path. If node *z* belongs to a flow path *f*, z∈f is recorded. Let For (z,f) and Bac(z,f) denote the sets of the forward and backward nodes of a flow path *f*, respectively. For example, as shown in Figure 2a, For(R3, $f_{1}$) = {D1}, Bac(R3, $f_{1}$) = {R1, R2, S1}, For(R3,$f_{2}$) = {R4, R5, D2}, and Bac(R3, $f_{2}$) = {S2}. Moreover, when *n* flows $\left(f_{1},f_{2},\cdots ,f_{n}\right)$ intersect at node *c*, the definitions of the primary coding conditions are specified as follows:

**Definition** **1.** *Assuming that any two flows $f_{a}$ and $f_{b}$ pass in any intersection node c, the necessary and sufficient conditions of coding opportunities are as follows:*

*1.*  Existing node $d_{1}$∈ Bac($c,f_{a}$), meanwhile $d_{1}$∈$N(s_{2})$ and $s_{2}$∈ For($c,f_{b}$), or $d_{1}$∈ Bac($c,f_{b}$).
*2.*  Existing node $d_{2}$∈ Bac($c,f_{b}$), meanwhile $d_{2}$∈$N(s_{1})$ and $s_{1}$∈ For($c,f_{a}$), or $d_{2}$∈ Bac($c,f_{a}$).

Given that existing backward nodes (i.e., $d_{1}\in$ Bac($c,f_{a}$) and $d_{2}\in$ Bac($c,f_{b}$)) must overhear or have the original packets in the primary coding conditions, this can ensure that the destinations have decoding opportunities and obtain their respective packets. Moreover, $s_{1}$, $s_{2}$ and $d_{1}$, $d_{2}$ can be arranged with any node along the backward and forward nodes of the flow instead of the source and destination nodes [12,14,15]. Moreover, the intermediate nodes need to decode the encoded packets at the earliest possible moment to guarantee the decoding success ratio. For example, take the dotted box in Figure 2a. Node R4 is the selected node, which can receive the encoded Packet P1⊕P2 and overhear the original packets from Node R2. Thus, based on the primary coding conditions, Node R4 will decode the encoded Packet P1⊕P2 and transmit the original Packet P2. However, Node R4 will be closed to the coding Node R3. The decoding and coding nodes will gather in the region. The network load will be congested and have an unbalanced distribution, especially in more complex networks.

Then, we propose the constrained coding conditions to trade off the network load and coding opportunities. The definition of the constrained coding conditions is specified as follows:

**Definition** **2.** *Assuming that any two flows $f_{a}$ and $f_{b}$ pass in any intersection node c, the necessary and sufficient conditions of coding opportunities are as follows:*

*1.*  Existing node $d_{1}$∈ Bac($c,f_{a}$), meanwhile $d_{1}$∈ {$N(s_{2})$∩ Bac($c,f_{a}$)} and $s_{2}$∈ For($c,f_{b}$), or $d_{1}$∈ Bac($c,f_{b}$).
*2.*  Existing node $d_{2}$∈ Bac($c,f_{b}$), meanwhile $d_{2}$∈ {$N(s_{1})$∩ Bac($c,f_{b}$)} and $s_{1}$∈ For($c,f_{a}$), or $d_{2}$∈ Bac($c,f_{a}$).

In the constrained coding conditions, $s_{1}$, $s_{2}$ and $d_{1}$, $d_{2}$ are still the source and destination nodes. Moreover, the decoding nodes can be near the destination node. Then, the network hot-spots can be avoided. As shown in Figure 2b, Node R5 is the decoding node and the neighbor node next to D2 and S1, which is away from the coding Node R3.

**Figure 3:**
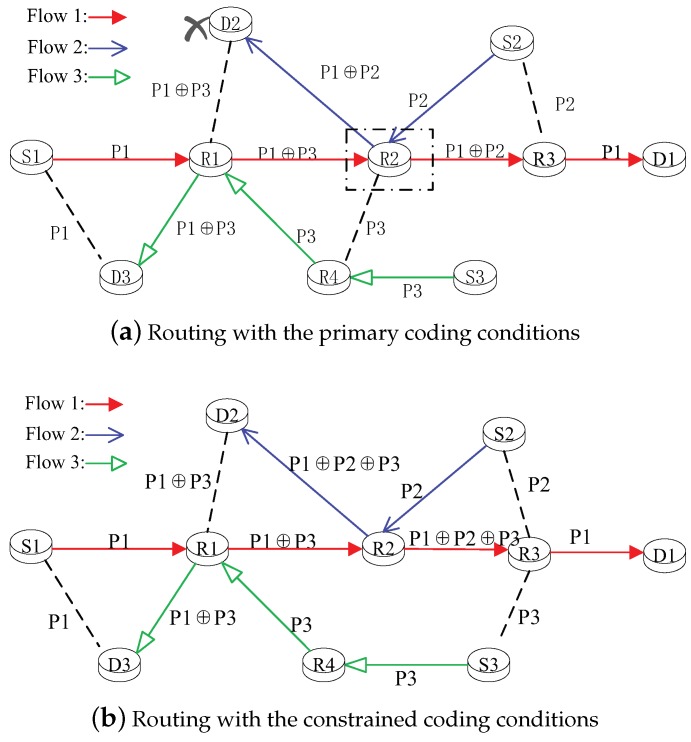
The routing decision with different coding conditions in a multi-flow network.

In a multi-flow network, the primary coding conditions can also lead to the re-encoding phenomenon and decrease the decoding-success ratio. For example, three flows exist at a certain moment in Figure 3, (i.e., $f_{1}$ (from Node S1 to Node D1), $f_{2}$ (from Node S2 to Node D2), and $f_{3}$ (from Node S3 to Node D3)). As shown in Figure 3a, because the flows $f_{1}$ and $f_{3}$ satisfy the primary coding conditions, Node R1 will be selected as a coding node and broadcast the encoded Packet P1⊕P3. Node R2 can receive the encoded Packet P1⊕P3. To prevent subsequent no-decoding opportunities in the future, the encoded packet should be decoded as soon as possible. Thus, Node R2 decodes the encoded Packet P1⊕P3 and generates the original Packet P1. However, flows $f_{2}$ and $f_{3}$ meet at Node R2, and Node R2 also has a coding opportunity to encode the original Packets P1 and P2. To this end, Node R2 transmits the encoded Packet P1⊕P2. Node R2 contains both the coding and decoding opportunities. The burden of Node R2 will increase, and the network load will be unbalanced. Moreover, Node D2 only overhears the encoded packets P1⊕P3, and it cannot decode the encoded Packet P1⊕P2 received from Node R2. To avoid these phenomena, we can use the constrained coding conditions to judge the coding opportunities. As shown in Figure 3b, similar to the primary coding conditions, Node R1 is also the coding node for flows $f_{1}$ and $f_{3}$. However, Node R2 continues to encode the received Packet P1⊕P3 with the original Packet P2 and generates the encoded Packet P1⊕P2⊕P3. The decoding node is selected as Node R3 instead of Node R2, which can overhear the original packets P2 and P3. We can see that the decoding node is constrained in the neighbors of the source node to avoid the hot-spot problem. In addition, the destination Node D2 can successfully decode the received Packet P1⊕P2⊕P3 due to it being able to overhear the encoded Packet P1⊕P3 from Node R1. We can see that the constrained coding conditions can avoid the re-encoding problem and ensure the decoded success probability.

Moreover, compared with the constrained coding conditions, the primary coding conditions have a higher computational complexity. They often need to calculate each node of the forward and backward flows and loop twice. Then, we analyze the computational complexity in a two-flow network, which is a simple scene and similar to that of multi-flow networks. In a two-flow network, we define that the numbers of the forward nodes of the flows $f_{1}$ and $f_{2}$ are $J_{1}$ and $J_{2}$, respectively, and the numbers of the backward nodes of flows $f_{1}$ and $f_{2}$ are $K_{1}$ and $K_{2}$, respectively. The computational complexity is $O\left(J_{1}K_{2}+J_{2}K_{1}\right)=O\left(n^{2}\right)$. However, the constrained coding conditions only need to calculate the number of neighbor nodes of the sources, and the sources are known. We assume that the number of neighbor nodes of Sources S1 and S2 are $L_{1}$ and $L_{2}$, respectively, and the computational complexity is $O\left(L_{1}+L_{2}\right)=O\left(n\right)$.

### 3.3. Routing Determination

#### 3.3.1. Routing and Coding Discovery Processes

In the opportunity-routing mechanisms, which CAR belongs to, the routing and coding discovery processes are on-demand. In addition, the processes are often launched via the RREQ (route request) messages by the source nodes and replied via to the RREP (route reply) messages by the destination nodes in practice. Some researchers have extended the routing discovery method by using an extra handshake (routing confirm and routing acknowledge) to ensure the encoding success ratio and enhance the decoded success ratio. It works well, but is not efficient in a large-scale network [15]. Moreover, the routing and coding discovery processes were separately operated in the CAR mechanisms, which would cause more energy consumption [38].

In this paper, we adopted the traditional method, which only uses RREQ and RREP messages. Through the routing request and reply processes, we can also find the available paths for the data flow and detect the potential coding opportunities of the paths simultaneously, termed the “routing + coding” discovery process.

**Figure 4:**
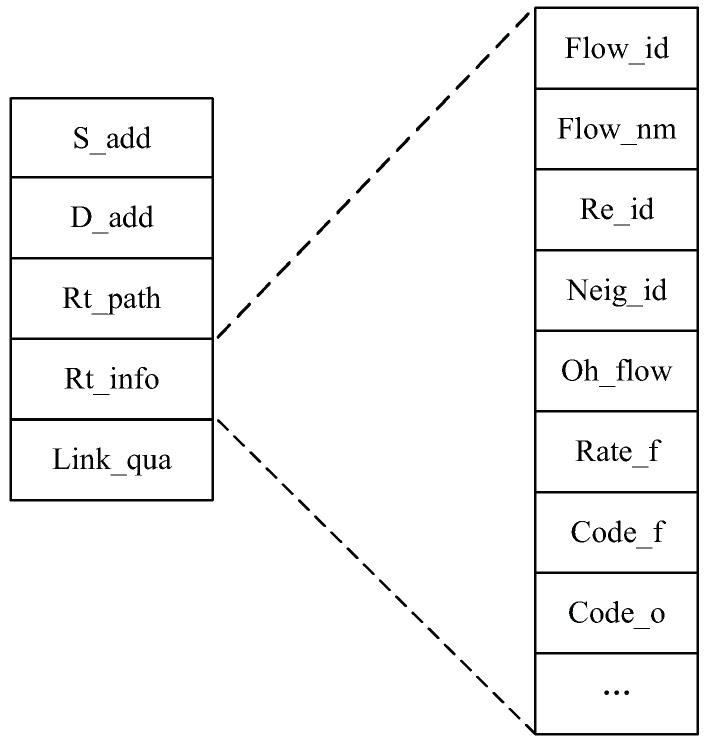
The format of the RREP message header.

To be suitable for the “routing + coding” discovery process, we modify the structure of the RREP message, which is shown in Figure 4. S_add and D_add denote the addresses of the source and destination nodes, respectively. Rt_path records the routing path that has been passed. Furthermore, Rt_info denotes the route information. Rt_info consists of the flow IDs (Flow_id), the number of flows (Flow_nm), and the relay node IDs (Re_id). Moreover, it includes neighbor information (e.g., the addresses of the neighbor nodes (Neig_id) and the overheard flows (Oh_flow)) to help judge the state of routes and detect the coding opportunities according to Definitions 1 and 2. Rate_f denotes the flow rate. The coding flows and coding opportunities are marked as Code_f and Code_o, respectively. The end of the RREP message is Link_qua, which denotes the link quality (i.e., packet loss probability).

**Figure 5:**
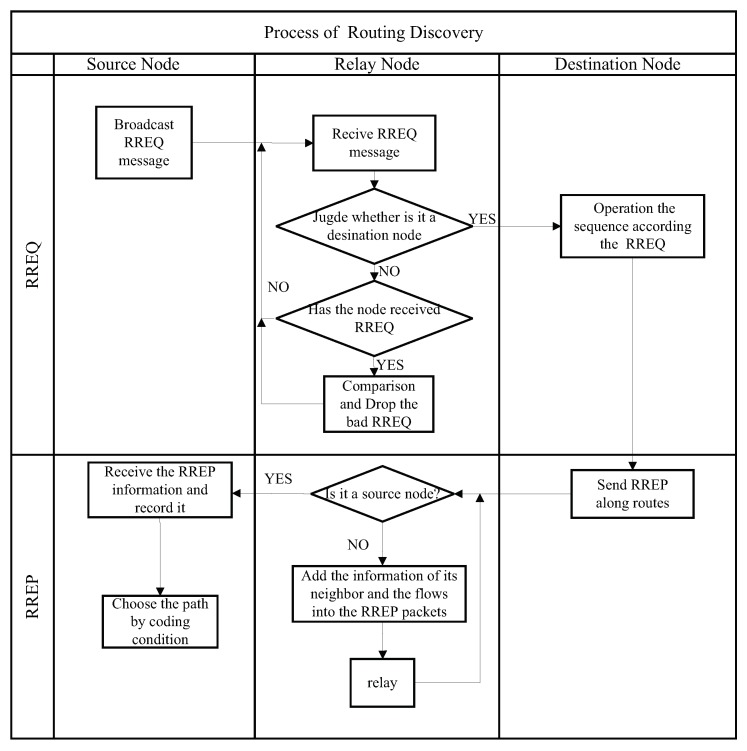
The flowchart of routing discovery.

When a new flow arrives at the wireless network, the source node of the new flow activates the “routing + coding” discovery process using the RREQ and RREP messages. Moreover, the flowchart of routing discovery is summarized as shown in Figure 5.

Then, we simply describe the function of the RREQ and RREP messages as follows:

- RREQ (routing request message)
  The RREQ message is a simplified version of the RREP message, which contains the source and destination addresses, the relay node IDs, the overheard flows, the link qualities, and other pertinent information. To start with the “routing + coding” discovery process, the source node broadcasts the RREQ messages at the moment a new flow appears. When the RREQ message arrives at a relay node, the node will calculate the link quality (Link_qua) and determine to which node it should be forwarded. If the relay node has received some RREQ messages that have the same destination, it will compare the link qualities of those messages. Then, the relay node will temporarily store one RREQ message that has the best link quality and drop the others. After the destination node has received the RREQ messages from different relay nodes in a fixed period, it can sort all candidate transmission routes according to the information (e.g., link quality and transmission counts) in the RREQ messages and then choose the best route.  
- RREP (routing reply message)
  The RREP message should be transmitted to the source node through a candidate route. When the RREP message arrives at a node, the node will verify the information in the RREP message (i.e., S_add and D_add). If the node is not the destination, it adds the route information of the intersected flows, the overheard flows, and one-hop neighbors into the RREP message. The relay nodes will forward the RREP message to the source node through unicasting. Moreover, the link quality between the relay node and its next hop is recorded in the RREP message.

#### 3.3.2. An Illustrative Example

To clearly demonstrate how to discover the “routing + coding” processes, we present a simple example within the topology given in Figure 2b. Assuming that Flow 1 (S1-R1-R2-R3-D1) is an existing flow, we want to find a path for new Flow 2 (S2-D2). The process proceeds as follows.

1. In the initiation of the discovery process, Node S2 broadcasts the RREQ message.
2. When Node R3 receives the RREQ message, it first checks whether the RREQ message has already arrived. If so, Node R3 needs to discard the redundant RREQ message to prevent the loop. If not, Node R3 will temporally store the “ who-can-overhear” information (i.e., Nodes R2 and R4 can overhear the flow from Node R3) and the upper path (i.e., the upper path is S2-R2). Node R3 then updates its overhear information (i.e., adding Nodes R2, R4, and D1 into the neighbor list) and broadcasts the RREQ message.
3. When one or more RREQ messages arrive at destination Node D2 in a limited time, D2 sorts all received RREQ messages, then automatically chooses the available paths with the best link quality, and inserts the inverse of the path into the RREP message.
4. When Node R2 receives these RREP messages, it will compare the path with its temporarily-stored information. Moreover, according to the constrained coding conditions, Node R2 has the coding opportunity in which Flow 2 can be encoded with existing Flow 1, and the RREP message will mark the node as a coding node.
5. As the RREP message returns to the source Node S2, based on the information of potential coding opportunities and routing coding benefit (which will be described in Section 3.4 in detail), the source node will decide the final route.

For example, the routing information of Flow 2 is shown in Table 3. It is stored in the RREP message to help the sources determine the best route. Moreover, the size of each part in Table 3 can be modified according to different situations. In this experiment, the total storage overhead is 184 bits in this table, which is large enough to store the route information.

### 3.4. Routing Coding Benefit

In the available paths, we need to choose the best path. Therefore, we design a novel routing metric to quantify all the available paths in this subsection. The existing routing metrics are roughly divided into two categories to measure the routing coding benefit (i.e., topology-based metrics and load-based metrics). In the CAR schemes, topology-based metrics include the three following measures. (1) hop count: it is often used because of its simple application. It only counts the hop numbers of the link and ignores the link quality and other link characteristics. (2) ETX(the expected transmission count) [39]: it can calculate the number of expected successful transmissions and reflect the link quality. (3) ETT (the expected transmission time) [40]: it is extended from ETX and includes the link bandwidth and flow rate. The load-based metrics often take into account the current interfering traffic in the routing decision. In [41], B. Ni et al. proposed an expected number of coded transmission (ECX) metric that considered the number of encoded packets, the channel capacity, and the packet loss probability. H. Chen et al. proposed a group-aware cooperative routing metric (GAR) that applies the group feature and the packet delivery probability to reduce the network load in resource-constrained networks [42]. However, most load-based metrics are too difficult to implement and have high costs. The coding-aware routing metric (CRM) was proposed by J. Le et al. [10], which takes into account the encoded queue length. The CRM showed that the estimation of the queue length consumed less power. Moreover, in [37], we discussed the queue state of the coding nodes with the Markov model. In this paper, we propose a new metric called expected transmission count based on queue length (EXQL) that combines the topology-based and load-based metrics to choose a good path. EXQL consists of two sections in which (1) a coding benefit function is used to calculate the coding gain of the encoded packets by the routing information, and (2) based on the queue length, a weight factor is used to reflect the state of the coding node. In addition, the EXQL metric can adjust to the constrained coding conditions and simplify the routing implementation.

#### Coding Benefit Function with Queue Length

Data transmission with NC can reduce the transmission number and improve the performance in the multi-hop scenario [6]. Similar to ETX, we utilized the minimum expected transmission count to mainly measure the coding benefit. Before we introduce the coding benefit function, the symbols should be defined. Assume that *k* candidate routes exist and $P_{l}\left(1\leq l\leq k\right)$ denotes the $l^{\mathrm{th}}$ candidate path. Let $H(P_{l})$ be the hop number of the $l^{\mathrm{th}}$ candidate path. The minimum expected number among the set of all the candidate paths can be defined as follows:

(1) $$e_{\min}\left(P_{l}\right)=\min_{1\leq l\leq k}H\left(P_{l}\right).$$

We can see that the hop number of transmissions along the candidate paths is always larger than or equal to $e_{\min}$. Moreover, the path cost $M(P_{l})$ can be defined as:

(2) $$M\left(P_{l}\right)=H\left(P_{l}\right)-e_{\min}\left(P_{l}\right).$$

Then, considering that two packets are coded as shown in Figure 1b, one transmission can be saved. Analogously, if *n* packets are encoded in a coding node, n−1 transmissions can be saved due to the broadcast. While there are $n_{m}$ packets in the coding node $C_{m}$ on a candidate path, the benefit of the coding node $C_{m}$ can be calculated as:

(3) $$\beta \left(C_{m}\right)=n_{m}-1.$$

As there are *v* coding nodes in the candidate path $P_{l}$, we define $D(P_{l})$ as the benefit of candidate path $P_{l}$, which can be shown as follows:

(4) $$D\left(P_{l}\right)=\sum_{m=1}^{v}\beta \left(C_{m}\right).$$

According to (1), (2), and (4), to compute the actual number of transmissions in a multi-flow network on each path, we define the candidate path benefit $\beta (P_{l})$ as follows:

(5) $$\begin{array}{cc} \beta (P_{l}) & =D\left(P_{l}\right)-M\left(P_{l}\right)\\ & =\sum_{m=1}^{v}\left(n_{m}-1\right)-\left[H\left(P_{l}\right)-e_{\min}\left(P_{l}\right)\right]. \end{array}$$

We can see that $\beta (P_{l})$ contains the benefit of decreased hop number $D(P_{l})$ and path cost $M(P_{l})$. Equation (5) indicates that we should choose the shorter path with more coding benefits. The larger the path benefit $\beta (P_{l})$ is, the better the path. Moreover, the path benefit can be easily calculated when $e_{\min}$ is selected and when the network is large.

**Figure 6:**
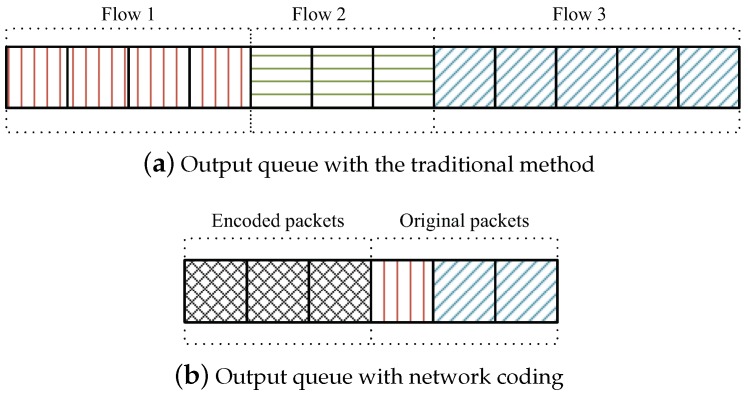
An example of the output queue.

When multiple flows with different rates merge on a coding node, the coding node will be in a confused state, and the coding opportunity may be bad. Moreover, the state of the coding nodes is an important factor to determine the network delay [34,35,37]. Therefore, we need to consider the state of the coding nodes. It is important to note that the queue length is more feasible to calculate [10,37]. For a realistic scenario with processes with different rates, each flow with different rates has different numbers of input queues in a time-slot. In this scheme, to resolve the multi-rate matching problem, instead of the accurate and complex measurement, we adopt an approximate method (i.e., using the queue length) to estimate the state of coding nodes. In the coding nodes, the output queue will be encoded and shortened by NC. For example, as shown in Figure 6, it is assumed that a coding node receives 4, 3, and 5 original packets from Flows 1, 2, and 3, respectively. In the traditional method, the total output queue length is 12. However, by the NC technology, the output queue length is shortened to six, and the length of encoded packets is three. Moreover, when the encoded packets are in the output queue, the coding nodes can transmit the unencoded at the same time, which is the free-rider phenomenon. In the output queue of coding nodes, more encoded and fewer original packets show that the coding opportunity is good. Therefore, we can obtain the proportion of the number of encoded packets in the output queue to measure the state of coding nodes.

Assume that *t* flows intersect in the coding node $C_{m}$. We define $L(f_{j})$ as the length of input queue of flow j(1≤j≤t). Moreover, in the coding node $C_{m}$, we define that $L(O_{e})$ is the length of the output queue, and $L(O_{n})$ is the length of encoded packets in the output queue. The encoded packets’ occupancy proportion $\frac{L(O_{n})}{L(O_{e})}$ shows the coding ability and state. A larger value of occupancy proportion shows more encoded packets in the output queue and better coding effects. Moreover, in a perfect situation, the output queue may be all encoded packets, for which the length of encoded packets will be approximately $\left\lfloor \frac{\sum_{j=1}^{t}L\left(f_{j}\right)}{t}\right\rfloor$. To this end, we use a weight α ( 0<α≤1) to react to the state of coding node $C_{m}$ as follows:

(6) $$\alpha \left(C_{m}\right)=\left\{\begin{array}{cc} 1 & \left\lfloor \frac{\sum_{j=1}^{t}L\left(f_{j}\right)}{t}\right\rfloor \geq L\left(O_{e}\right)\\ \frac{L(O_{n})}{L(O_{e})} & \left\lfloor \frac{\sum_{j=1}^{t}L\left(f_{j}\right)}{t}\right\rfloor <L\left(O_{e}\right) \end{array}\right..$$

The coding benefit in a multi-rate scenario can be recalculated as $\beta_{r}\left(C_{m}\right)=\alpha \left(C_{m}\right)\beta \left(C_{m}\right)=\alpha \left(C_{m}\right)\left(n_{m}-1\right)$ by combining Equations (3) and (6). $D_{r}\left(P_{l}\right)$ represents the benefit of candidate path $P_{l}$, which can be recalculated as $D_{r}\left(P_{l}\right)=\sum_{m=1}^{z}\beta_{r}\left(C_{m}\right)$. Then, we can define the EXQL metric as follows:

(7) $$\begin{array}{cc} EXQL(P_{l}) & =D_{r}\left(P_{l}\right)-M\left(P_{l}\right)\\ & =\sum_{m=1}^{v}\beta_{r}\left(C_{m}\right)-M\left(P_{l}\right)\\ & =\sum_{m=1}^{v}\left[\alpha \left(C_{m}\right)\beta \left(C_{m}\right)\right]-M\left(P_{l}\right) \end{array}$$

In the scheme, considering the impact of the state of coding nodes, we used the EXQL metric to calculate the coding benefit of each candidate route. The sources can transmit the packets through a better route path to reduce the negative impact of multi-rate and -flow in the coding nodes.

### 3.5. Encoding and Decoding Implementation

**Figure 7:**
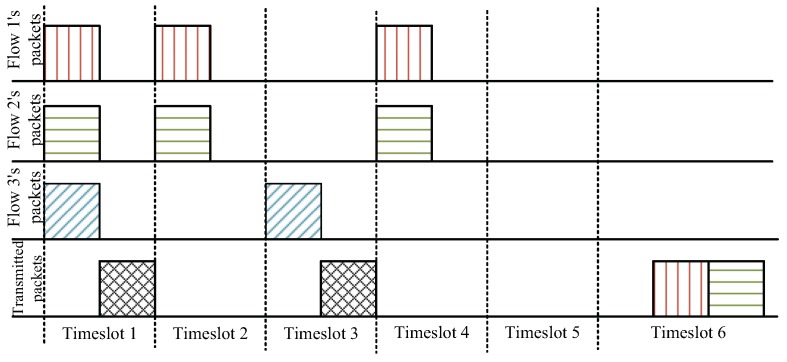
An example of the encoding implementation.

In this subsection, we discuss the issue of the encoding implementation involved in the constrained coding-aware routing scheme. To facilitate the analysis, we define a fixed duration *T*. In this scheme, we used the time division duplex (TDD) technique to divide each timeslot *T* into two sub-timeslots T/2. TDD is one of the duplexing methods and applied to the IEEE 802.11g standard, which is widely used in wireless ad-hoc networks. The duplexing methods allow receiving and sending at the same time. The primary benefit of TDD is that only one channel is needed. It saves spectrum space and cost. Moreover, it is possible to change the uplink and downlink capacity ratio dynamically according to the need. Moreover, TDD requires time synchronization at the network level. IEEE 802.11 standards specify a clock synchronization protocol (i.e., the network time protocol (NTP)), and the related content can be found in [43,44]. In the first sub-timeslot, the relay nodes receive the original packets. Moreover, the encoded packets are generated by original packets and transmitted in the second sub-timeslot. As shown in Figure 7, in Timeslot 1, the node receives three original packets and transmits an encoded packet in different sub-timeslots.

Because the coding nodes receive the original packets from different flows with different rates, the coding node needs an additional timeslot to await coding opportunities. We present a simple example with a one packet/timeslot flow rate, which is as shown in Figure 7. The coding opportunities are to encode three packets from Flows 1, 2, and 3, and the original packets are successfully encoded in Timeslot 1. In Timeslot 2, the packets from Flows 1 and 2 dissatisfy the preset coding opportunities. The coding node needs an extra timeslot (e.g., Timeslot 3) to wait for another packet from Flow 3 and temporarily puts the received packets into the packet pool. In addition, notice that the coding node does not spend too much time waiting for the slow rate flows. In addition, in this paper, we set the expiration time to two timeslots. For example, in the two timeslots 4–5, the coding node cannot encode the original packets, and the node should directly transmit the originals in Timeslot 6. This encoding implementation not only guarantees coding opportunities, but also reduces the network delay.

Let $a_{j}\left[x\right]$ denote the packets from flow *j* in the output queue of coding node $C_{m}$ at the end of the $x^{\mathrm{th}}$ timeslot. Then, $a_{j}\left[x\right]$ can be determined as:

(8) $$a_{j}\left[x\right]=a_{j}\left[x-1\right]+b_{j}\left[x\right]-d_{j}\left[x\right],$$

where $a_{j}\left[x-1\right]$ denotes the packets awaiting delivery at the $x-1^{\mathrm{th}}$ timeslot and $b_{j}\left[x\right]$ denotes the original packets from flow *j*, which is also the input queue from flow *j*. $d_{j}\left[x\right]$ denotes the packets that can be encoded in the input queue at the end of the $x^{\mathrm{th}}$ timeslot. If *t* flows intersect in coding node $C_{m}$, the encoded packets $d_{m}\left[x\right]=⊕d_{j}\left[x\right]\;\left(1\leq j\leq t\right)$. We also denote the output queue of the coding node $C_{m}$ as $U_{m}\left[x\right]$ at the end of the $x^{\mathrm{th}}$ timeslot, which is given by:

(9) $$U_{m}\left[x\right]=\sum_{j=1}^{t}a_{j}\left[x\right]+d_{m}\left[x\right].$$

According to the previous analysis, we present an encoding algorithm as shown in Algorithm 1. When a coding node has no coding opportunity (i.e., code_o ≠ T), it will proceed to Step 2. If it has a coding opportunity (i.e., code_o = T), it will examine whether the arriving flows can be encoded according to the constrained coding conditions as Step 5 or 9. When the arriving flows are less than the required number (i.e., flow_n < code_f), no packets are placed in the output queue $U_{m}\left[x\right]$ as Step 6, and the expire time of coding will be set to two timeslots as Step 7. Steps 9–17 show the detailed process of encoding the packets of different sizes. More specifically, we first find the minimum packet length mi of $b_{j}\left[x\right]$, which can be encoded in the input queue. Then, we select mi packets from each $b_{j}\left[x\right]$ and put these packets in $d_{j}\left[x\right]$. The node encodes $d_{j}\left[x\right]$ and generates $d_{m}\left[x\right]$ (i.e., $d_{m}\left[x\right]=⊕d_{j}\left[x\right]\;\left(1\leq j\leq t\right)$). To this end, push $d_{m}\left[x\right]$ and $\sum_{j=1}^{t}a_{j}\left[x\right]$ into $U_{m}\left[x\right]$ according to Equation (9).

**Algorithm 1** Encoding algorithm in each timeslot.**Input:**$b_{j}\left[x\right]$ code_o, code_f **Output:**
$U_{m}\left[x\right]$1:**while** code_o ≠ T **do**2:  $U_{m}\left[x\right]=\sum_{j=1}^{t}b_{j}\left[x\right]$3:**end while** 4:**while** code_o = T **do**5:  **if** flow_n < code_f **then**6:    $U_{m}\left[x\right]=0$ 7:    Return Expire_time = 2 timeslots 8:  **end if** 9:  **if** flow_n = code_f **then**10:    Statistic mi = $\min b_{j}\left[x\right]$  11:    **for** n=1,n<mi,n++ **do**
12:      Select mi packet from each $b_{j}\left[x\right]$, and put these packets in $d_{j}\left[x\right]$ 13:      $d_{m}\left[x\right]=⊕d_{j}\left[x\right]\;\left(1\leq j\leq t\right)$ 14:      Push $d_{m}\left[x\right]$ into $U_{m}\left[x\right]$ 15:      Push $\sum_{j=1}^{t}a_{j}\left[x\right]$ into $U_{m}\left[x\right]$ 16:    **end for** 17:  **end if** 18:**end while** 19:**return**$U_{m}\left[x\right]$  

The decoding implementation is simple and the reverse process of encoding. The node uses the encoded and original packets from the buffer pool for proper decoding. Each packet has its own packet ID, which is stored in a hash table. The table is collected every few seconds. When a node wants to recover an encoded packet consisting of *j* original packets, the node goes through the IDs of the *j* original packets one by one and retrieves the corresponding packet from its packet pool if possible. To this end, the encoded packet XORs the j−1 original packets, which can recover an original packet. Notice that the lost encoded packet and original packets should be retransmitted to help decode. Moreover, each packet in the buffer pool is assigned with a long enough packet timeout.

## 4. Performance Evaluation

To evaluate the performance of the proposed CCAR scheme, we used the ns-2 simulator, which is a discrete event simulator widely used in network research. In the following experiments, all nodes are in the promiscuous mode and time synchronized. We adopted IEEE 802.11g in the MAC layer. In addition, we used the user datagram protocol (UDP) traffic source and constant bit rate (CBR) flows with a fixed packet size of 512 bytes. The packet loss ratio was set to 5%. The radio transmission range was 25 m. The flow rate was set to 100–400 kbps. The maximum length of the input and output queue and packet pool was uniformly set to 200 packets, which was long and large enough on each node. The packet in the packet pool would be purged when the packet pool was full. Moreover, each node can maintain a table of routing information and process a list of one-hop neighbors using periodical exchanging of the HELLO messages. Periodical exchanging of HELLO messages has been widely used by some CAR schemes, and it will not impose more message overhead [10,14,15]. Our simulation results were averaged over five randomly-generated simulations, and the topologies were created by the “setdest” tool in the ns-2 simulation. In addition, we chose two different topologies to evaluate the different performances: one is a grid topology, and the other is a random topology. It is worth noting that the time and energy consumed by the data process can be ignored because the XOR operation is simple and fast, and the time and energy consumed by data processing is less than that consumed by communicating the data.

### 4.1. Results from the Grid Topology

**Figure 8:**
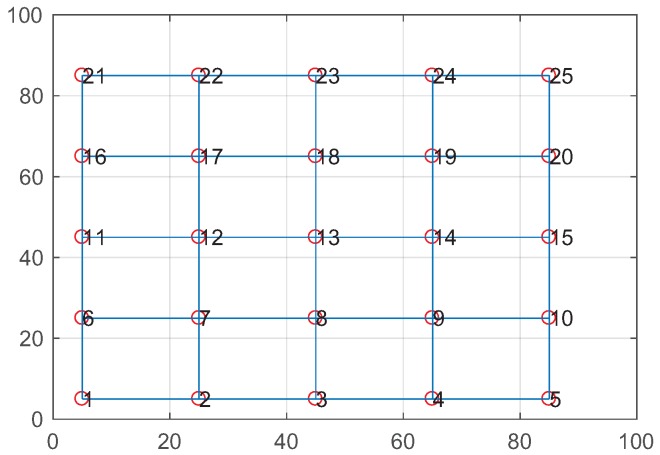
A 5 × 5 grid topology.

To avoid the impact of topology on performance, we analyzed the effective coding benefit, the constrained coding conditions, and the EXQL metric in the grid topology. We used a 5 × 5 grid topology as shown in Figure 8, in which two adjacent nodes are separated 20 m vertically and horizontally. Eight sources were randomly generated, and each generated flow had a randomly-picked source, destination, and flow rate. There were 2–5 hops between each source and destination node.

#### 4.1.1. Effective Coding Benefit

In this experiment, we first analyzed the encoded and decoded packet ratio of the schemes. The encoded packet ratio measures the number of encoded packets in proportion to the total number of transmitted packets. The decoded packet ratio is the proportion of the number of successfully-decoded packets to the total number of the encoded packets.

**Figure 9:**
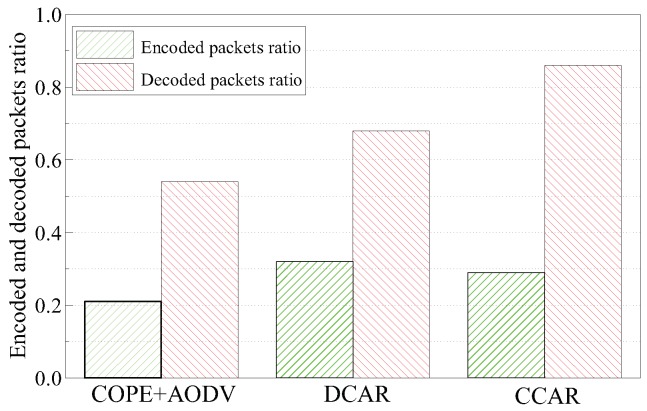
Encoded and decoded packet ratios.

Figure 9 shows the comparison of the encoded and decoded packet ratios of the three protocols (i.e., COPE + AODV, DCAR, and CCAR). We can see that the encoded packet ratio of DCAR was the highest, exceeding those of CCAR by 10% and AODV + COPE by 52% in the 5 × 5 grid topology, because the DCAR mechanism had the most coding opportunities. However, the encoded packet ratio only represents the encoding ability. In the case of the multi-flow interference, not all encoded packets can be successfully decoded. Then, we considered the decoded packet ratio. Compared to DCAR and COPE + AODV, CCAR had the best decoded opportunity, being approximately 26% better than DCAR and 59% better than AODV + COPE, since it provided more accurate decoding by the constrained coding conditions.

**Figure 10:**
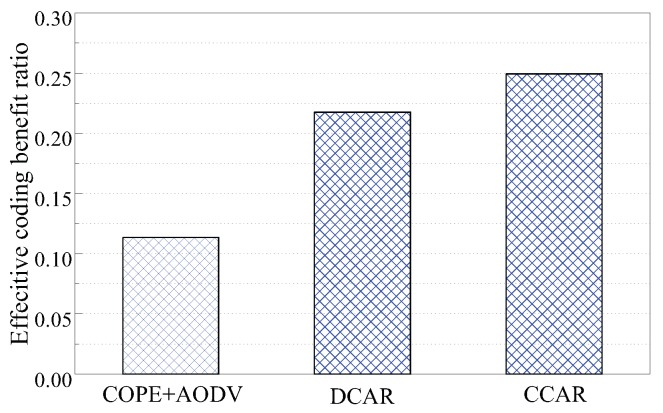
Effective coding benefit ratio.

We then analyzed the effective coding benefit ratio, which shows the successfully-decoded packets in the overall transmitted packets. It is defined by the product of the encoded packet ratio and the decoded packet ratio. The effective coding benefit ratio can comprehensively reflect the abilities of coding and decoding. Figure 10 presents the effective coding benefits of the three protocols under the grid topology. The benefit of CCAR is better than for the other schemes because CCAR has the highest decoded packet ratio.

#### 4.1.2. The Impact on Coding Conditions and Coding Benefit Function

By comparing arbitrary combinations of different coding conditions (CCC and PCC) and coding benefit functions (EXQL and ETX), we can comprehensively evaluate the performance of each coding condition and coding benefit function. To judge the performance in the multi-rate scenario, a flow was selected with different flow rates.

**Figure 11:**
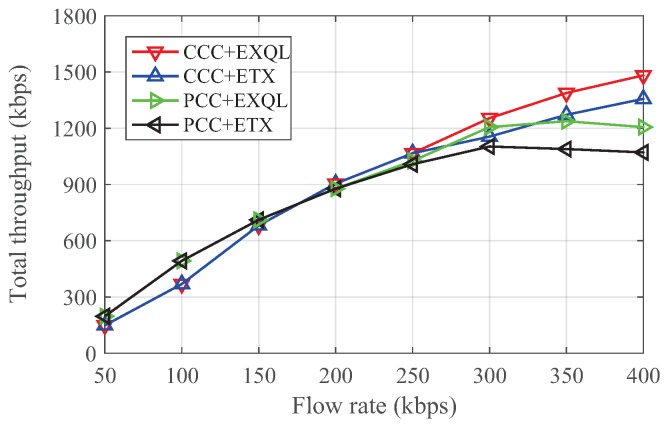
Total throughput versus flow rate in the grid network. EXQL, expected transmission count based on queue length.

**Figure 12:**
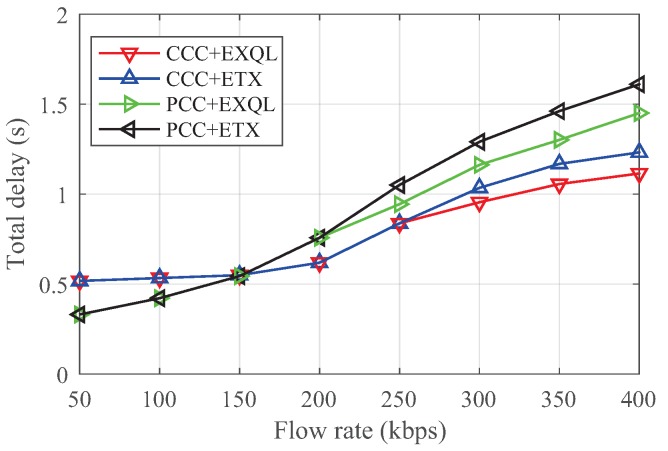
Delay versus flow rate in the grid network.

Figure 11 and Figure 12 show the total throughput and total delay under different rates. When the rate was less than 150 kbps, the total throughput of PCC was better than for CCC, and the delay time of PCC was weaker than for CCC, because PCC had more coding opportunities. Moreover, given the lower rate and fewer number of packets transmitted, the network was not too congested. ETX and EXQL had similar performances in the case of a low flow rate. However, when using a flow rate greater than 200 kbps, CCC could achieve better throughput and less delay time than PCC, while the network state deteriorated. According to the judgment of the state of the coding node, EXQL also performed better than ETX in a more congested network. This result also verifies that the coding node state had a large impact on the performance.

### 4.2. Results from a Random Topology

**Figure 13:**
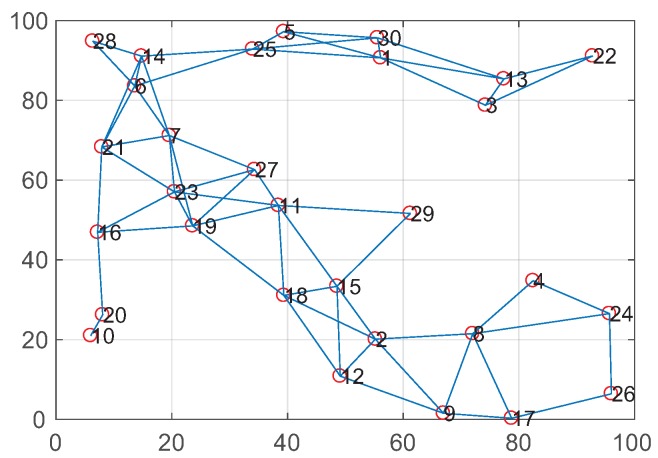
A random topology with 30 nodes.

The other scenario is a random topology, which is a more complex network. We constructed a random topology with 30 nodes in 100 × 100 m${}^{2}$ areas, as shown in Figure 13, and the numbers of neighbors in each node were 1–6. Moreover, we compared the performance of CCAR, DCAR, and AODV + COPE under the random topology and evaluated the total throughput, average end-to-end delay, and energy consumption under different numbers of flows from 4–16.

#### 4.2.1. The Impact on Throughput

**Figure 14:**
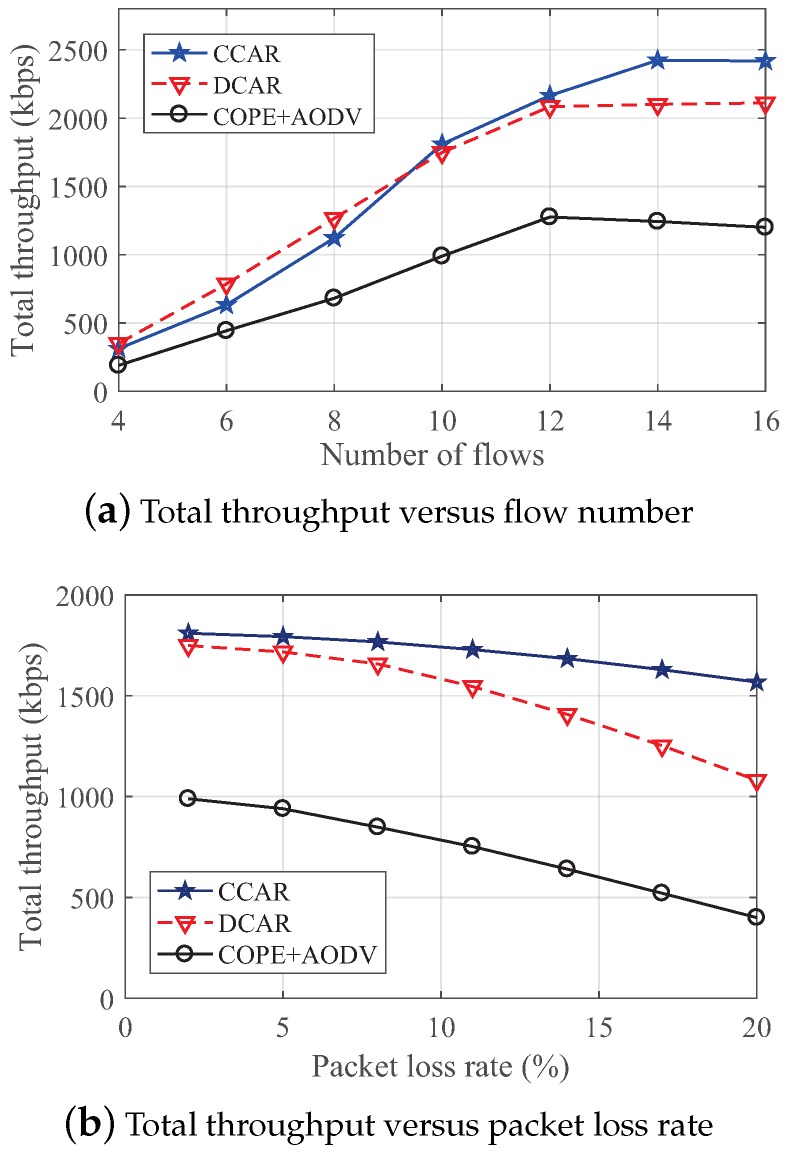
The impact on total throughput in the random network.

Figure 14a shows the total throughput of these three schemes under different numbers of flows. As the number of flows increased, the throughput of all the schemes gradually increased. However, when the number of flows exceeded 12, the throughput of DCAR and COPE + AODV tended to flatten and descend. This was because congestion appeared with the increased payload. In addition, the throughput of CCAR reached a peak value at 14 flows and began to stabilize. This shows that CCAR had the best robustness to the network congestion. It is worth noting that the throughput of DCAR was larger than CCAR when the number of flow was less than eight, implying that DCAR can obtain more coding opportunities in a small-scale network.

In addition, the total throughput is exhibited under different packet loss rates with 10 flows in Figure 14b. We can observe that the total throughput of those three schemes decreased when the packet loss rate increased. CCAR had the largest throughput and best robustness compared to the other two schemes. This performance was due to the constrained coding condition that can guarantee decoding opportunities. Moreover, the value of DCAR declined fastest because the scheme had more encoded packets that could not be successfully decoded.

#### 4.2.2. The Impact on Average End-to-End Delay

**Figure 15:**
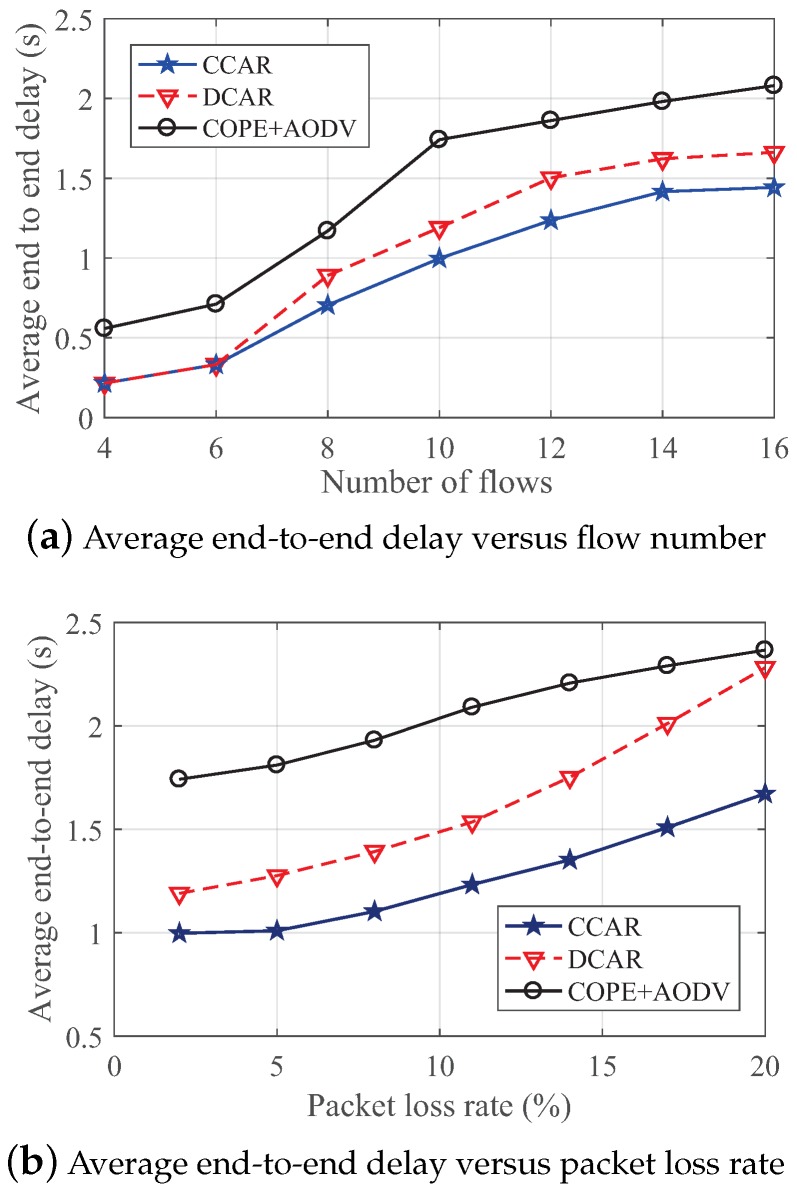
The impact on average end-to-end delay in the random network.

The average end-to-end delay under different numbers of flows in the random topology is investigated in Figure 15a. The average end-to-end delay can show the effectiveness of these three protocols on a single route. Not surprisingly, CCAR had a smaller delay than for DCAR and COPE + AODV because our scheme can select a better route while considering the state of coding nodes, which significantly influences the delay.

Moreover, Figure 15b presents the average end-to-end delay with different packet loss rates in 10 flows network. Similar to Figure 14b, we can see that the proposed CCAR scheme had the best robust performance with average end-to-end delay. When the packet loss rate was at a high level, DCAR declined fastest and was close to COPE + AODV. This is because DCAR is sensitive to the packet loss rate. The primary coding conditions in DCAR had a poor effect in a high packet loss rate scenario.

#### 4.2.3. The Impact on Energy Consumption

Next, we investigated the energy consumption under different numbers of flows in the random topology. Energy consumption was mainly decided by the physical transmission distance [45]. Therefore, to facilitate analysis and calculate the energy consumption, we employed a widely-used simplified model shown as follows:

(10) $$\begin{array}{c} E_{T}\left(y,g\right)=E_{c}\times y+\xi_{amp}\times y\times g^{2}, \end{array}$$

(11) $$\begin{array}{c} E_{R}\left(y\right)=E_{c}\times y, \end{array}$$

where $E_{T}\left(y,g\right)$ denotes the energy consumed for transmitting *y*-bits of data over the Euclidean distance *g*. $E_{R}\left(y\right)$ represents the energy consumed for receiving *y*-bits of data. $E_{c}$ is a constant value that denotes the energy consumed for transmitting or receiving a one-bit message. $\xi_{amp}$ is the transmission power amplification. In addition, we set the constant values $E_{c}=100$ nJ/bit and $\xi_{amp}=200$ pJ/bit/m${}^{2}$ in the simulation.

**Figure 16:**
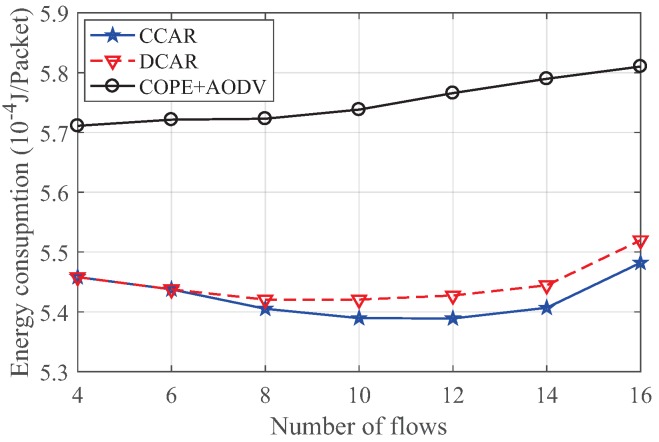
Energy consumption versus flow number in the grid network.

The energy consumed per packet under different numbers of flows is given in Figure 16. The energy consumed under the COPE + AODV scheme smoothly and slightly rose with the increase in the number of flows. Moreover, we can see that the CCAR and DCAR schemes had similar trends. In particular, in the case of a low number of flows, both schemes had the same energy consumption. As the number of flows became large, the energy consumption decreased first in the range of 4–16 because the network coding can encode packets and decrease the number of transmissions to counteract the effects of multiple flows. Moreover, for a larger number of flows, the advantages of CCAR began to emerge, and its energy consumption was lowest because the proposed scheme had the highest decoded packet ratio and reduced the re-transmission process.

## 5. Conclusions

In this paper, we reviewed the shortcomings of coding-aware routing in multi-flows and multi-rate networks and proposed a constrained coding-aware opportunistic routing (CCAR) to increase the performance of the multi-flow and multi-rate networks. The proposed mechanism consists of three components:

1. It contains the constrained coding conditions to avoid the hotspot and solve the re-encoded problem.
2. To suit the constrained coding conditions, we added some routing information (such as Flow_nm, Neig_id and Code_f) in the RREP messages and simplified the process of routing and coding discovery.
3. In the EXQL metric, the novel coding benefit function introduces a weight factor that consists of queue length to resolve the rate matching problem and reduce the network delay.

The simulation results for the grid topology demonstrated that the constrained coding conditions and the EXQL metric can improve the success ratio of decoded packets and trade off coding opportunities. In the random topology, the proposed CCAR scheme can significantly improve performance in terms of the network throughput and the average end-to-end delay compared with the existing DCAR and AODV + COPE schemes.

In future work, we will further consider a more harsh scenario with a higher packet loss rate, node mobility, and the limitations of buffer size. 

## Figures and Tables

**Table 1:** Overview of the key NC-aware routing protocols. DCAR, distributed coding-aware routing; FROM, free-ride-oriented routing metric.

NC-aware Protocol	Coding Type	Routing Metric	Algorithm Type	Network Coding Range	Evaluation Scenario
COPE [5]	Inter-flow	Coding gain	Centralized	Two-hop	Wireless multi-hop networks
CAOR [9]	Inter- and	ETX	Centralized	Two-hop	Lossy wireless networks
	intra-flow				
DCAR [10]	Inter-flow	CRM	Distributed	Multi-hop	Wireless ad hoc networks
FROM [12]	Inter-flow	FORM	Distributed	Multi-hop	Wireless mesh backbone networks
LFCR [13]	Inter-flow	RPC	Distributed	Multi-hop	Wireless multi-hop networks
CFCR [14]	Inter-flow	CDS	Distributed	Multi-hop	Wireless mesh networks
DGCDR [15]	Inter-flow	DGCDR	Distributed	Multi-hop	Wireless multi-flow networks
I2NC [27]	Inter- and	NUM	Centralized	Two-hop	Wireless mesh networks
	intra-flow				
HyCare [34]	Inter-flow	N-ETOX	Distributed	Multi-hop	Wireless multi-hop networks

**Table 2:** Definition of terms.

Terms	Definition
Original packets	Nonencoded data packets
Encoded packets	The packets after the encoded operation
Encoded flows	The flows that incorporate the encoded packets
Coding nodes	The nodes that meet the coding conditions and perform the encoded function
Decoding nodes	The nodes that can decode the encoded packets
Output queue	A queue at each node to deposit the packets that need to be sent

**Table 3:** The routing information stored in the RREP message.

Flow (4b)	*f*_2_				
Hops (4b)	4				
Path (32b)	S1	R3	R4	R5	D2
Neighbors (32b)	D1	-	R2	S1	-
Overheard flows (32b)	$f_{1}$	$f_{1}$		$f_{1}$	-
Coding opportunities (16b)	-	-	T	-	-
Coding flows (16b)	-	-	$f_{1}$	-	-
Flow rate (32b)	$R_{f_{2}}$	$R_{f_{2}}$	$R_{f_{1}}$,$R_{f_{2}}$	$R_{f_{2}}$	$R_{f_{2}}$
Link quality (16b)	$Q_{1},Q_{2},Q_{3},Q_{4}$				
